# Cancer related adverse events associated with use of proton pump inhibitors and histamine-2 receptor antagonists: A real-world analysis using the FDA adverse event reporting system

**DOI:** 10.1371/journal.pone.0329385

**Published:** 2025-08-12

**Authors:** Bowen Wang, Zheyun Song, Shan Lan, Xiubi Chen, Xida Yan, Xue-Feng Jiao

**Affiliations:** 1 Department of Rehabilitation Medicine, West China Second University Hospital, Sichuan University, Chengdu, China; 2 Xinxiang Medical University, Xinxiang, China; 3 Sichuan Center for Food and Drug Evaluation, Inspection & Monitoring, SCFDA Adverse Drug Reaction Monitoring Center Medical Device Technology Review and Evaluation Center, Chengdu, China; 4 Center for Adverse Drug Reaction Monitoring of Mianyang, Mianyang, China; 5 Department of Pharmacy, Mianyang Central Hospital, Mianyang, China; 6 Department of Pharmacy/Evidence-Based Pharmacy Center, West China Second University Hospital, Sichuan University; Children’s Medicine Key Laboratory of Sichuan Province, Chengdu, China; 7 NMPA Key Laboratory for Technical Research on Drug Products In Vitro and In Vivo Correlation, Chengdu, China; 8 Key Laboratory of Birth Defects and Related Diseases of Women and Children, Sichuan University, Ministry of Education, Chengdu, China; University of Sindh, PAKISTAN

## Abstract

Despite widespread use, concerns have emerged regarding the increased risks of digestive system cancers associated with use of proton pump inhibitors (PPIs). Moreover, ranitidine was recalled from the market in some countries due to its potential carcinogenicity, which also has raised concern about cancer risk in association with use of histamine-2 receptor antagonists (H2RAs). We comprehensively explored the potential risks of various cancers associated with use of PPIs and H2RAs by analyzing the FDA Adverse Event Reporting System (FAERS), aiming to offer real-world evidence for the safe and rational use of acid suppressive agents. OpenVigil 2.1 was utilized to query the FAERS database. Cancer related adverse events (AEs) included Preferred Terms (PTs) of malignant neoplasms among all cancer sites. Disproportionality analysis was performed, and a positive signal indicated a statistical association between cancer related AEs and drugs. Most PPIs had more cancer related PTs with positive signals than H2RAs (except ranitidine), but had fewer cancer related PTs with positive signals than ranitidine. Forty-three cancer related PTs exhibited positive signals for more than one PPIs, and the major cancer sites of these PTs were gastric, lung, lymphomas, pancreatic, oesophageal, intestinal, upper respiratory tract, renal, soft tissue, and so on. Besides, only two cancer related PTs exhibited positive signals for more than one H2RAs (except ranitidine). Our study suggests that PPIs may be associated with more cancer related AEs than H2RAs (except ranitidine), but may be associated with fewer cancer related AEs than ranitidine. Except for digestive system cancers, use of PPIs may also be associated with increased risks of multiple non-digestive system cancers. According to our findings, H2RAs (except ranitidine) may be safer than PPIs regarding cancer risk, and the priority use of PPIs for acid suppression therapy may not be appropriate.

## Introduction

Acid-suppressive agents, including proton pump inhibitors (PPIs) and histamine-2 receptor antagonists (H2RAs), are extensively utilized in treating gastrointestinal disorders such as peptic ulcers, dyspepsia, gastroesophageal reflux disease, upper gastrointestinal bleeding, and Zollinger-Ellison syndrome. Due to their stronger acid suppression and more favorable safety profile, PPIs are generally preferred over H2RAs, prompting many patients to switch from H2RA therapy to PPIs [1,2]. Consequently, PPIs are now among the most commonly prescribed medications worldwide [3].

Concerns regarding the potential cancer risk associated with PPIs have emerged as their use has become widespread. Several clinical studies have associated PPI use with a higher risk of digestive system cancers, such as gastric [4], colorectal [5], pancreatic [6], esophageal [7], and liver cancers [8], compared to H2RA use. These findings suggest that H2RAs might offer a safer profile concerning cancer risk, raising questions about the preferential use of PPIs for acid suppression therapy.

In recent years, however, ranitidine—a widely used H2RA—was recalled in the United States, Europe, and Japan due to elevated levels of the probable human carcinogen N-nitrosodimethylamine (NDMA) [9,10]. Furthermore, several clinical studies have linked ranitidine use with an increased risk of gastrointestinal and bladder cancers [11–13]. These findings have intensified concerns about the carcinogenic risks associated with H2RAs.

The FDA’s Adverse Event Reporting System (FAERS) ranks among the world’s largest and most respected spontaneous-reporting databases, housing more than 25 million adverse – event submissions and offering a detailed view of medication safety in real – world practice [14]. Advanced data-mining methods have been applied to FAERS to flag “positive signals,” which denote statistically significant links between drugs and reported events [15]. Given its extensive sample size, FAERS has the statistical capability to detect rare adverse drug reactions that might be overlooked in conventional epidemiologic studies, making it a key resource for exploring potential adverse drug reaction signals [16–22]. Therefore, in this analysis, we comprehensively explored the potential risks of various cancers associated with use of PPIs and H2RAs by analyzing the FAERS database, aiming to offer real-world evidence for the safe and rational use of acid suppressive agents.

## Methods

### Data source

FAERS is a spontaneous reporting system that aids the FDA in post-marketing surveillance of approved drugs and therapeutic biological products in the United States. The publicly accessible FAERS database includes demographic details, administrative records, drug and adverse event information, source reports, and patient outcomes [23]. Since the data does not contain identifiers for patients or reporters, it precludes access to any information that could link reports to individual participants. We queried the FAERS database using OpenVigil 2.1 (https://openvigil.sourceforge.net/), a pharmacovigilance tool developed for data extraction, cleaning, and analysis [24,25]. This study examined FAERS data from Q1 2004 to Q1 2022.

### Identifying PPIs and H2RAs

OpenVigil 2.1 standardizes drug names—encompassing brand names, generic names, and abbreviations—by mapping them to a unique identifier using data from Drugs@FDA and Drugbank. We first identified unique identifiers for each PPI (omeprazole, lansoprazole, rabeprazole, pantoprazole, esomeprazole, dexlansoprazole and ilaprazole) and H2RA (ranitidine, cimetidine, famotidine, nizatidine and roxatidine) using the “Browse Window”. We searched the drug names of PPIs and H2RAs (both drugs as a class and individual drugs) using the “OpenVigil Search Window”.

### Definition of cancer related AEs

In FAERS, AEs were coded adopting Preferred Terms (PTs) in the Medical Dictionary for Regulatory Activities (MedDRA) terminology. In our analysis, cancer related AEs included PTs of malignant neoplasms among all cancer sites [26].

### Statistical analysis

OpenVigil 2.1 was utilized for conducting the disproportionality analysis. The “Data Presentation and Statistics Box” calculated the proportional reporting ratio (PRR) to evaluate the statistical association between cancer related AEs and drugs. A higher PRR signifies a stronger association, with a PRR of two indicating that the AE is two times more frequent in the users of a target drug than in the background population. Following the criteria outlined by Evans et al. (2001), a positive signal of disproportionality is identified by the occurrence of three or more cases, a PRR of two or greater, and a chi-squared value of four or higher [27].

## Results

### Summary of AE reports for PPIs and H2RAs

Table 1 provides a summary of AE reports for PPIs submitted to FAERS. The numbers of AE reports for PPIs as a class, omeprazole, lansoprazole, rabeprazole, pantoprazole, esomeprazole, dexlansoprazole and ilaprazole were 493197, 205911, 107138, 23054, 140927, 151497, 31931 and 27, respectively. A substantial proportion of these AE reports had missing gender or age information. Without considering missing information, more females than males reported AEs for all types of PPIs except ilaprazole, and most AE reports occur in the 41–65 and over 65 age groups. The primary reporting country was the United States, and the most common serious outcome in these reports was hospitalization. Following disproportionality analysis, the numbers of cancer related PTs with positive signals for PPIs as a class, omeprazole, lansoprazole, rabeprazole, pantoprazole, esomeprazole, dexlansoprazole and ilaprazole were 96, 56, 42, 28, 59, 38, 6 and 0, respectively.

**Table 1:** Summary of AE reports for PPIs submitted to FAERS.

	PPIs as a class	Omeprazole	Lansoprazole	Rabeprazole	Pantoprazole	Esomeprazole	Dexlansoprazole	Ilaprazole
**Number of AE reports (n, %)**	493197	205911	107138	23054	140927	151497	31931	27
**Gender** (n, %)								
Male	170167 (34.50%)	68354 (33.20%)	31059 (28.99%)	6604 (28.65%)	46352 (32.89%)	40557 (26.77%)	5082 (15.92%)	24 (88.89%)
Female	255110 (51.73%)	98041 (47.61%)	42692 (39.85%)	10156 (44.05%)	64251 (45.59%)	72976 (48.17%)	9014 (28.23%)	3 (11.11%)
Unknown	67920 (13.77%)	39516 (19.19%)	33387 (31.16%)	6294 (27.30%)	30324 (21.52%)	37964 (25.06%)	17835 (55.85%)	0
**Age** (n, %)								
0–17 years	6442 (1.31%)	2993 (1.45%)	1753 (1.64%)	115 (0.50%)	777 (0.55%)	1078 (0.71%)	24 (0.08%)	0
18–40 years	29143 (5.91%)	11742 (5.70%)	4690 (4.38%)	1143 (4.96%)	6555 (4.65%)	6935 (4.58%)	628 (1.97%)	2 (7.41%)
41–65 years	137865 (27.95%)	51298 (24.91%)	21379 (19.95%)	5741 (24.90%)	34173 (24.25%)	40418 (26.68%)	4159 (13.02%)	23 (85.19%)
>65 years	136786 (27.73%)	49383 (23.98%)	22973 (21.44%)	6028 (26.15%)	37108 (26.33%)	28756 (18.98%)	2184 (6.84%)	2 (7.41%)
Unknown	182961 (37.10%)	90495 (43.95%)	56343 (52.59%)	10027 (43.49%)	62314 (44.22%)	74310 (49.05%)	24936 (78.09%)	0
**Reported Countries (Top five ranked)**								
United States	299051 (60.64%)	141303 (68.62%)	68836 (64.25%)	12582 (54.58%)	86362 (61.28%)	121914 (80.47%)	30198 (94.57%)	0
United Kingdom	42796 (8.68%)	23875 (11.59%)	16002 (14.94%)	455 (1.97%)	1781 (1.26%)	1786 (1.18%)	14 (0.04%)	0
Canada	22886 (4.64%)	2761 (1.34%)	1984 (1.85%)	2710 (11.76%)	11788 (8.36%)	3394 (2.24%)	1298 (4.07%)	16 (59.26%)
France	19896 (4.03%)	3754 (1.82%)	2815 (2.63%)	818 (3.55%)	5939 (4.21%)	7117(4.70%)	0	0
Germany	18448 (3.74%)	3193 (1.55%)	204 (0.19%)	73 (0.32%)	14231 (10.10%)	1085 (0.72%)	0	0
**Serious outcomes**								
Hospitalization	162952 (33.04%)	57510 (27.93%)	26695 (24.92%)	6922 (30.03%)	48633 (34.51%)	31066 (20.51%)	2050 (6.42%)	9 (33.33%)
Death	40087 (8.13%)	17087 (8.30%)	9649 (9.01%)	3225 (13.99%)	13230 (9.39%)	10534 (6.95%)	2886 (9.04%)	0
Life-threatening	20956 (4.25%)	7690 (3.73%)	3861 (3.60%)	939 (4.07%)	5430 (3.85%)	3536 (2.33%)	176 (0.55%)	1 (0.04%)
Disability	14524 (2.94%)	6011 (2.92%)	3341 (3.12%)	722 (3.13%)	2635 (1.87%)	2726 (1.80%)	250 (0.78%)	0
**Number of cancer related PTs with positive signals** (n)	96	56	42	28	59	38	6	0

AE, adverse event; PPIs, proton pump inhibitors; FAERS, FDA Adverse Event Reporting System; PTs: Preferred Terms. These data were obtained through querying and analyzing the FAERS database using OpenVigil 2.1 (https://openvigil.sourceforge.net/).

Table 2 provides a summary of AE reports for H2RAs submitted to FAERS. The numbers of AE reports for ranitidine, H2RAs as a class (except ranitidine), cimetidine, famotidine, nizatidine and roxatidine were 363728, 36051, 4244, 31364, 1058 and 5, respectively. Similarly, a substantial proportion of these AE reports had missing gender or age information. Without considering missing information, more females than males reported AEs for cimetidine, famotidine and nizatidine, whereas more males than females reported AEs for ranitidine and roxatidine. Moreover, most AE reports occur in the 41–65 and over 65 age groups. The primary reporting country was the United States, and the most common serious outcome in these reports was hospitalization. Following disproportionality analysis, the numbers of cancer related PTs with positive signals for ranitidine, H2RAs as a class (except ranitidine), cimetidine, famotidine, nizatidine and roxatidine were 162, 24, 10, 24, 1 and 0, respectively.

**Table 2:** Summary of AE reports for H2RAs submitted to FAERS.

	Ranitidine	H2RAs as a class (except ranitidine)	Cimetidine	Famotidine	Nizatidine	Roxatidine
**Number of AE reports** (n, %)	363728	36051	4244	31364	1058	5
**Gender** (n, %)						
Male	161542 (44.41%)	13172 (36.54%)	1391 (32.78%)	11585 (36.94%)	410 (38.75%)	3 (60.00%)
Female	142803 (39.26%)	19958 (55.36%)	2496 (58.81%)	17178 (54.77%)	579 (54.73%)	2 (40.00%)
Unknown	59383 (16.33%)	2921 (8.10%)	357 (8.41%)	2601 (8.29%)	69 (6.52%)	0
**Age** (n, %)						
0–17 years	2772 (0.76%)	1019 (2.83%)	81 (1.91%)	911 (2.90%)	29 (2.74%)	0
18–40 years	15492 (4.26%)	2816 (7.81%)	381 (8.98%)	2400 (7.65%)	58 (5.48%)	0
41–65 years	116586 (32.05%)	10241 (28.41%)	1452 (34.21%)	8670 (27.64%)	340 (32.14%)	1 (20.00%)
>65 years	53601 (14.74%)	9502 (26.36%)	1020 (24.03%)	8234 (26.25%)	330 (31.19%)	3 (60.00%)
Unknown	175277 (48.19%)	12473 (34.60%)	1310 (30.87%)	11149 (35.55%)	301 (28.45%)	1 (20.00%)
**Reported Countries (Top five ranked)**						
United States	292795 (80.50%)	25973 (72.05%)	2770 (65.27%)	23199 (73.97%)	581 (54.91%)	0
United Kingdom	5078 (1.40%)	561 (1.56%)	214 (5.04%)	260 (0.83%)	88 (8.32%)	0
Canada	3202 (0.88%)	387 (1.07%)	135 (3.18%)	240 (0.77%)	17 (1.61%)	0
France	673 (0.19%)	137 (0.38%)	97 (2.29%)	43 (0.14%)	2 (0.19%)	0
Germany	735 (0.20%)	116 (0.32%)	59 (1.39%)	57 (0.18%)	0	0
**Serious outcomes**						
Hospitalization	18562 (5.10%)	11681 (32.40%)	1320 (31.10%)	10063 (32.08%)	416 (39.32%)	2 (40.00%)
Death	21765 (5.98%)	3572 (9.91%)	466 (10.98%)	3096 (9.87%)	89 (8.41%)	1 (20.00%)
Life-threatening	2436 (0.67%)	1733 (4.81%)	258 (6.08%)	1413 (4.51%)	62 (5.86%)	0
Disability	3244 (0.89%)	1050 (2.91%)	184 (4.34%)	840 (2.68%)	57 (5.39%)	0
**Number of cancer related PTs with positive signals** (n)	162	24	10	24	1	0

AE, adverse event; H2RAs, histamine-2 receptor antagonists; FAERS, FDA Adverse Event Reporting System; PTs: Preferred Terms. These data were obtained through querying and analyzing the FAERS database using OpenVigil 2.1 (https://openvigil.sourceforge.net/).

### Positive signals for PPIs

**Fig 1:**
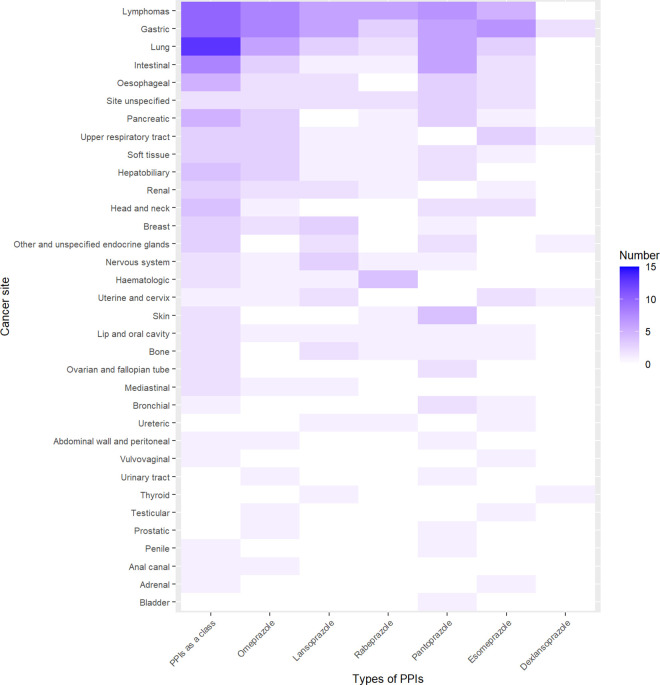
Number of cancer related PTs with positive signals in each cancer site for PPIs. PTs, Preferred Terms; PPIs, proton pump inhibitors.

For PPIs as a class, positive signals emerged in 96 cancer related PTs, as detailed in S1 Table. The major cancer sites of these 96 PTs were lung, gastric, lymphomas, intestinal, pancreatic, oesophageal, hepatobiliary, head and neck, upper respiratory tract, renal, breast, and soft tissue (Fig 1 and S13 Table).

For omeprazole, positive signals emerged in 56 cancer related PTs, as detailed in S2 Table. The major cancer sites of these 56 PTs were gastric, lymphomas, lung, intestinal, pancreatic, hepatobiliary, upper respiratory tract, soft tissue, oesophageal, renal, and breast (Fig 1 and S13 Table).

For lansoprazole, positive signals emerged in 42 cancer related PTs, as detailed in S3 Table. The major cancer sites of these 42 PTs were gastric, lymphomas, lung, breast, nervous system, oesophageal, renal, uterine and cervix, and bone (Fig 1).

For rabeprazole, positive signals emerged in 28 cancer related PTs, as detailed in S4 Table. The major cancer sites of these 28 PTs were lymphomas, haematologic, gastric, and lung (Fig 1 and S13 Table).

For pantoprazole, positive signals emerged in 59 cancer related PTs, as detailed in S5 Table. The major cancer sites of these 59 PTs were lymphomas, gastric, intestinal, lung, skin, pancreatic, oesophageal, hepatobiliary, bronchial, ovarian and fallopian tube, head and neck, and soft tissue (Fig 1 and S13 Table).

For esomeprazole, positive signals emerged in 38 cancer related PTs, as detailed in S6 Table. The major cancer sites of these 38 PTs were gastric, lymphomas, upper respiratory tract, lung, intestinal, oesophageal, uterine and cervix, and head and neck (Fig 1 and S13 Table).

For dexlansoprazole, positive signals emerged in 6 cancer related PTs, as detailed in S7 Table. The major cancer site of these 6 PTs was gastric (Fig 1 and S13 Table).

### Positive signals for H2RAs

**Fig 2:**
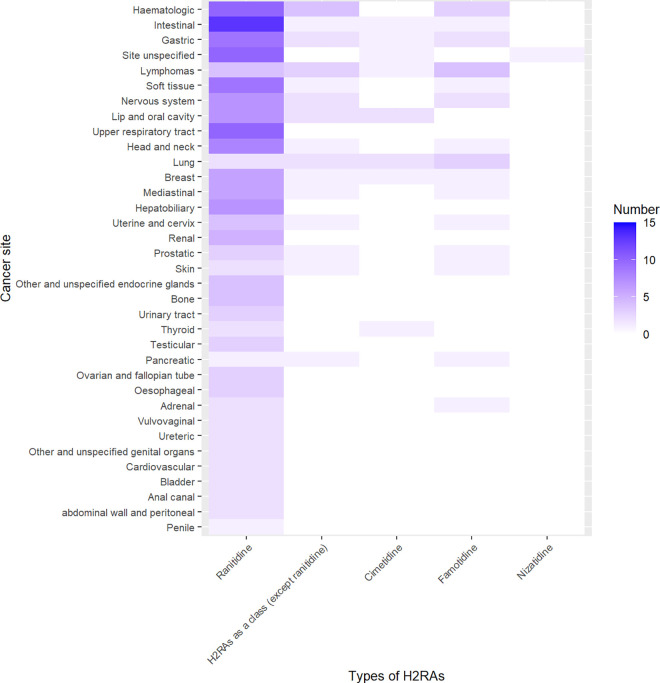
Number of cancer related PTs with positive signals in each cancer site for H2RAs. PTs, Preferred Terms; H2RAs, histamine-2 receptor antagonists.

For ranitidine, positive signals emerged in 162 cancer related PTs, as detailed in S8 Table. The major cancer sites of these 162 PTs were intestinal, upper respiratory tract, haematologic, gastric, soft tissue, head and neck, hepatobiliary, lip and oral cavity, nervous system, breast, mediastinal, renal, uterine and cervix, lymphomas, bone, oesophageal, urinary tract, prostatic, testicular, and ovarian and fallopian tube (Fig 2 and S14 Table).

For H2RAs as a class (except ranitidine), positive signals emerged in 24 cancer related PTs, as detailed in S9 Table. The major cancer sites of these 24 PTs were haematologic, lymphomas, gastric, lip and oral cavity, lung, and nervous system (Fig 2 and S14 Table).

For cimetidine, positive signals emerged in 10 cancer related PTs, as detailed in S10 Table. The major cancer sites of these 10 PTs were lip and oral cavity and lung (Fig 2 and S14 Table).

For famotidine, positive signals emerged in 24 cancer related PTs, as detailed in S11 Table. The major cancer sites of these 24 PTs were lymphomas, lung, haematologic, gastric, and nervous system (Fig 2 and S14 Table).

For nizatidine, positive signals emerged in one cancer related PTs, as detailed in S12 Table.

### Cancer related PTs exhibiting positive signals across multiple PPIs

A total of 43 cancer related PTs exhibited positive signals for more than one PPIs. The major cancer sites of these 43 PTs were gastric, lung, lymphomas, pancreatic, oesophageal, intestinal, upper respiratory tract, renal, and soft tissue. In addition, two of these PTs were identified as positive signal in six PPIs: adenocarcinoma gastric and metastatic gastric cancer. Five of these PTs were identified as positive signal in four PPIs: gastric neoplasm, gastrinoma, carcinoid tumour of the stomach, adenocarcinoma of colon, and bone neoplasm. Six of these PTs were identified as positive signal in three PPIs: gastrointestinal neoplasm, oesophageal adenocarcinoma, laryngeal neoplasm, lung adenocarcinoma stage III, nodal marginal zone B-cell lymphoma stage IV, and follicular lymphoma. Thirty of these PTs were identified as positive signal in two PPIs: adenomatous polyposis coli, adenocarcinoma pancreas, ductal adenocarcinoma of pancreas, pancreatic carcinoma metastatic, pancreatic neuroendocrine tumour, oesophageal cancer metastatic, oesophageal neoplasm, cholangiocarcinoma, lip neoplasm malignant stage unspecified, pharyngeal neoplasm, lung adenocarcinoma, lung neoplasm, non-small cell lung cancer stage IIIB, small cell lung cancer metastatic, squamous cell carcinoma of lung, bronchial neoplasm, carcinoid tumour, renal cell carcinoma stage IV, papillary renal cell carcinoma, ureteral neoplasm, transitional cell carcinoma, neoplasm prostate, ovarian cancer stage I, marrow hyperplasia, anaplastic large cell lymphoma T- and null-cell types, diffuse large B-cell lymphoma stage IV, hodgkin’s disease stage IV, Epstein-Barr virus associated lymphoma, spindle cell sarcoma, and mucoepidermoid carcinoma (Table 3).

**Table 3:** Cancer related PTs exhibiting positive signals across multiple PPIs.

Cancer site	PTs	Omeprazole	Lansoprazole	Rabeprazole	Pantoprazole	Esomeprazole	Dexlansoprazole
		PRR	PRR	PRR	PRR	PRR	PRR
Gastric	Adenocarcinoma gastric	12.100	13.165	12.476	17.783	8.984	7.850
Gastric	Metastatic gastric cancer	6.578	6.011	8.116	5.040	11.050	9.868
Gastric	Gastric neoplasm	4.903	2.777	—	3.820	4.731	—
Gastric	Gastrinoma	10.788	14.133	—	8.344	26.141	—
Gastric	Carcinoid tumour of the stomach	7.304	20.687	—	15.68	7.441	—
Gastric	Gastrointestinal neoplasm	2.117	—	6.911	—	2.734	—
Intestinal	Adenocarcinoma of colon	2.755	3.338	5.101	3.409	—	—
Intestinal	Adenomatous polyposis coli	6.742	—	—	8.344	—	—
Pancreatic	Adenocarcinoma pancreas	2.716	—	—	2.233	—	—
Pancreatic	Ductal adenocarcinoma of pancreas	3.480	—	—	5.114	—	—
Pancreatic	Pancreatic carcinoma metastatic	2.353	—	—	2.383	—	—
Pancreatic	Pancreatic neuroendocrine tumour	—	—	8.591	—	2.905	—
Oesophageal	Oesophageal adenocarcinoma	5.839	3.520	—	—	3.972	—
Oesophageal	Oesophageal cancer metastatic	5.208	—	—	3.109	—	—
Oesophageal	Oesophageal neoplasm	—	—	—	3.539	8.419	—
Hepatobiliary	Cholangiocarcinoma	2.289	—	—	2.014	—	—
Lip and oral cavity	Lip neoplasm malignant stage unspecified	2.756	—	—	—	4.911	—
Upper respiratory tract	Laryngeal neoplasm	2.720	—	—	—	9.292	8.687
Upper respiratory tract	Pharyngeal neoplasm	—	4.240	26.832	—	—	—
Lung	Lung adenocarcinoma stage III	4.373	—	—	26.423	15.627	—
Lung	Lung adenocarcinoma	—	2.152	2.481	—	—	—
Lung	Lung neoplasm	2.113	—	—	—	2.233	—
Lung	Non-small cell lung cancer stage IIIB	9.807	—	63.872	—	—	—
Lung	Small cell lung cancer metastatic	—	4.437	—	3.363	—	—
Lung	Squamous cell carcinoma of lung	—	3.463	—	—	2.439	—
Bronchial	Bronchial neoplasm	—	—	—	7.734	9.209	—
Other and unspecified endocrine glands	Carcinoid tumour	—	—	—	3.332	—	4.206
Renal	Renal cell carcinoma stage IV	7.245	—	—	—	4.093	—
Renal	Papillary renal cell carcinoma	6.915	26.894	—	—	—	—
Ureteric	Ureteral neoplasm	—	9.508	—	—	6.697	—
Urinary tract	Transitional cell carcinoma	2.032	—	—	2.831	—	—
Prostatic	Neoplasm prostate	2.345	—	—	4.818	—	—
Ovarian and fallopian tube	Ovarian cancer stage I	—	—	—	6.606	7.263	—
Haematologic	Marrow hyperplasia	—	3.214	4.014	—	—	—
Lymphomas	Nodal marginal zone B-cell lymphoma stage IV	215.751	418.344	—	—	294.680	—
Lymphomas	Follicular lymphoma	4.290	7.051	—	—	4.093	—
Lymphomas	Anaplastic large cell lymphoma T- and null-cell types	—	5.020	27.644	—	—	—
Lymphomas	Diffuse large B-cell lymphoma stage IV	4.731	3.486	—	—	—	—
Lymphomas	Hodgkin’s disease stage IV	—	—	35.831	5.800	—	—
Lymphomas	Epstein-Barr virus associated lymphoma	—	—	9.125	3.534	—	—
Bone	Bone neoplasm	—	2.967	6.849	3.125	3.180	—
Soft tissue	Spindle cell sarcoma	5.172	4.075	—	—	—	—
Soft tissue	Mucoepidermoid carcinoma	—	—	—	14.635	13.601	—

PTs, Preferred Terms; PPIs, proton pump inhibitors; PRR, proportional reporting ratio; —, not a positive signal. These data were obtained through querying and analyzing the FAERS database using OpenVigil 2.1 (https://openvigil.sourceforge.net/).

### Cancer related PTs exhibiting positive signals across multiple H2RAs (except ranitidine)

Two cancer related PTs exhibited positive signals for more than one H2RAs (except ranitidine): adenocarcinoma gastric and rectal cancer (Table 4).

**Table 4:** Cancer related PTs exhibiting positive signals across multiple H2RAs (except ranitidine).

Cancer site	PTs	Cimetidine	Famotidine
		PRR	PRR
Gastric	Adenocarcinoma gastric	25.058	11.528
Intestinal	Rectal cancer	6.452	2.846

PTs, Preferred Terms; H2RAs, histamine-2 receptor antagonists; PRR, proportional reporting ratio. These data were obtained through querying and analyzing the FAERS database using OpenVigil 2.1 (https://openvigil.sourceforge.net/).

## Discussion

### Main findings

To our knowledge, this study represents the most comprehensive exploration of the potential risks of various cancers associated with uses of PPIs and H2RAs. In our study, most PPIs had more cancer related PTs with positive signals than H2RAs (except ranitidine), but had fewer cancer related PTs with positive signals than ranitidine. The major cancer sites of PTs with positive signals for PPIs were gastric, lung, lymphomas, intestina, pancreatic, hepatobiliary, oesophageal, upper respiratory tract, renal, breast, soft tissue, and so on. The major cancer sites of PTs with positive signals for ranitidine were intestinal, upper respiratory tract, haematologic, gastric, soft tissue, head and neck, hepatobiliary, lip and oral cavity, nervous system, breast, mediastinal, renal, uterine and cervix, lymphomas, bone, and so on. The major cancer sites of PTs with positive signals for H2RAs (except ranitidine) were lymphomas, lung, gastric, haematologic, and so on. Moreover, 43 cancer related PTs exhibited positive signals for more than one PPIs, and 2 cancer related PTs exhibited positive signals for more than one H2RAs (except ranitidine).

### Explain the findings

The risks of digestive system cancers associated with use of PPIs have been noted previously. For example, a population-based cohort study using the UK Clinical Practice Research Datalink suggested that PPI therapy carried a 45% greater likelihood of gastric cancer versus H2RA use, with numbers needed to harm of 2121 after five years and 1191 after ten years post–treatment initiation [4]. Besides, a Taiwanese population cohort reported that PPI users faced a significantly higher colorectal cancer risk (adjusted hazard ratio [HR] 2.03; 95% confidence interval [CI] 1.56–2.63), which escalated with increased PPI exposure [5]. In another Taiwan‐based nested case-control study among patients with peptic ulcer disease or gastroesophageal reflux disease, PPI use was associated with greater odds of pancreatic cancer (adjusted odds ratio [OR] 1.69; 95% CI 1.44–2.05) [6]. In addition, a Swedish nationwide cohort found that long‐term PPI administration was linked to an almost fourfold rise in esophageal cancer incidence (standardized incidence ratio [SIR] 3.93; 95% CI 3.63–4.24) absent other risk factors [7]. Furthermore, both a nested case–control investigation using the Primary Care Clinical Informatics Unit database and a prospective UK Biobank cohort revealed that ever‐use of PPIs correlated with increased liver cancer risk (adjusted OR 1.80; 95% CI 1.34–2.41, and adjusted HR 1.99; 95% CI 1.34–2.94, respectively) [8]. Consistent with previous studies, our results also suggested that use of PPIs might be associated with increased risks of the above digestive system cancers. Therefore, the potential risks of digestive system cancers associated with use of PPIs should be given close attention in future clinical practice. The mechanisms underlying the increased risk of digestive system cancers associated with PPIs may involve several factors. First, PPIs can induce hypergastrinemia, which has been implicated in the development and progression of these cancers [28]. Additionally, the suppression of gastric acid secretion reduces the stomach’s natural defense against pathogenic bacteria, leading to increased colonization—particularly by nitrosamine-producing bacteria—which in turn may trigger chronic inflammation and promote carcinogenesis [29,30].

In our study, 43 cancer related PTs exhibited positive signals for more than one PPIs. Interestingly, except for digestive system cancers, the major cancer sites of these PTs also included lung, lymphomas, upper respiratory tract, renal, soft tissue, and so on. The positive associations between use of PPIs and the risks of these non-digestive system cancers (except prostate cancer) have not been reported or verified in previous epidemiologic studies [31]. However, as different types of cancers may share similar mechanism of pathogenesis, the potential risks of these non-digestive system cancers associated with use of PPIs warrant careful consideration in future clinical practice.

For H2RAs, our study found that ranitidine had much more cancer related PTs with positive signals than PPIs or other H2RAs, which were consistent with previous studies. Previous clinical studies also found that use of ranitidine is associated with increased risks of various cancers. For instance, a UK Biobank prospective cohort reported that habitual ranitidine users faced a 91% increase in liver cancer risk [11], while a nested case–control study in the Primary Care Clinical Informatics Unit database linked especially long‐term ranitidine use to heightened bladder cancer odds (adjusted ORs 1.22 [95% CI 1.06–1.40] and 1.43 [95% CI 1.05–1.94], respectively) [13]. These associations likely arise because ranitidine can degrade into high levels of N-nitrosodimethylamine (NDMA), a probable human carcinogen [11]. Although ranitidine has been withdrawn from markets in the United States, Europe, and Japan, it remains in use elsewhere (e.g., China), warranting continued caution and monitoring in clinical practice. Additionally, in our study, most cancer related PTs with positive signals emerged in ranitidine were not detected in other H2RAs, which seems that other H2RAs are safer than ranitidine regarding cancer risk. However, nizatidine has also been implicated in NDMA contamination [10]; our study included only 1058 AE reports for nizatidine, possibly limiting the power to detect signals. Therefore, additional large‐scale studies are needed to clarify any cancer risks associated with nizatidine.

Another important finding was that most PPIs had more cancer related PTs with positive signals than H2RAs (except ranitidine). Consistent with previous clinical studies, this finding also implies that H2RAs (except ranitidine) might be safer than PPIs regarding cancer risk, and does not support the priority use of PPIs for acid suppression therapy. The lower association of H2RAs (excluding ranitidine) with increased cancer risk compared to PPIs is not well understood, but it may be related to their weaker acid suppression. As H2RAs decrease acid suppression by blocking the effects of histamine only, they are less effective than PPIs and are less likely to induce hypergastrinaemia [32,33]. Further studies are still needed to both confirm this finding and explore the underlying mechanisms.

### Strengths and limitations

Our study has several notable strengths. First, with FAERS encompassing over 25 million adverse event reports, our analysis benefits from a sufficiently large sample size to detect rare adverse reactions. Second, by examining cancer related PTs across all cancer sites, we were able to conduct a comprehensive evaluation of the risks of various cancers associated with use of PPIs and H2RAs. Third, our work furnishes fresh, in-depth perspectives on the oncologic safety profiles of these acid‐suppressing drugs, informing more judicious clinical decision-making.

Besides, various methods, including reporting odds ratio (ROR), Bayesian confidence propagation neural network (BCPNN), and multi-item gamma Poisson shrinker (MGPS), have been developed to identify adverse drug reaction signals in FAERS. In this study, we used the standard of Evans et al. (2001) to detect adverse drug reaction signals, and this method is extensively utilized by the Medicines and Healthcare Products Regulatory Agency (MHRA). The standard of Evans et al. (2001) has higher specificity compared to the ROR method, and is significantly simpler to operate than the BCPNN and MGPS methods [34]. Thus, due to its advantages, the standard of Evans et al. (2001) is highly suitable for rapidly detecting a large number of cancer-related adverse reaction signals in our study.

Our study also has some limitations. First, the database is susceptible to under-reporting and duplicate entries, which may introduce reporting bias. Second, variations in the prevalence of specific PPIs and H2RAs could influence the number of reported AEs and the detected signals for different drugs, potentially impacting the comparability of cancer risk among them. Third, disproportionality analysis reveals only statistical associations and cannot establish causation. Therefore, further epidemiological studies are necessary to validate our results.

## Conclusion

Our analysis of the FAERS database suggests that PPIs may be associated with more cancer related AEs than H2RAs (except ranitidine), but may be associated with fewer cancer related AEs than ranitidine. Except for digestive system cancers, use of PPIs may also be associated with increased risks of cancers among lung, lymphomas, upper respiratory tract, renal, soft tissue, and so on. According to our findings, H2RAs (except ranitidine) may be safer than PPIs regarding cancer risk, and the priority use of PPIs for acid suppression therapy may not be appropriate. Moreover, ranitidine should be used cautiously due to its potential risk of developing many kinds of cancers. In addition, the potential risks of both digestive system cancers and non-digestive system cancers associated with use of PPIs warrant close attention in future clinical practice.

## Supporting information

S1 Table Cancer related AEs with positive signals for PPIs as a class. (DOCX)

S2 Table Cancer related AEs with positive signals for omeprazole. (DOCX)

S3 Table Cancer related AEs with positive signals for lansoprazole. (DOCX)

S4 Table Cancer related AEs with positive signals for rabeprazole. (DOCX)

S5 Table Cancer related AEs with positive signals for pantoprazole. (DOCX)

S6 Table Cancer related AEs with positive signals for esomeprazole. (DOCX)

S7 Table Cancer related AEs with positive signals for dexlansoprazole. (DOCX)

S8 Table Cancer related AEs with positive signals for ranitidine. (DOCX)

S9 Table Cancer related AEs with positive signals for H2RAs as a class (except ranitidine). (DOCX)

S10 Table Cancer related AEs with positive signals for Cimetidine. (DOCX)

S11 Table Cancer related AEs with positive signals for famotidine. (DOCX)

S12 Table Cancer related AEs with positive signals for nizatidine. (DOCX)

S13 Table Number of cancer related PTs with positive signals in each cancer site for PPIs. (DOCX)

S14 Table Number of cancer related PTs with positive signals in each cancer site for H2RAs. (DOCX)
