# Supplementary material for: Cancer related adverse events associated with use of proton pump inhibitors and histamine-2 receptor antagonists: A real-world analysis using the FDA adverse event reporting system
Source: PLoS One. 2025 Aug 12;20(8):e0329385. doi: 10.1371/journal.pone.0329385 (PMC12342331; doi:10.1371/journal.pone.0329385)
Supplement: S1 Table — (DOCX) [file pone.0329385.s001.docx]

**Supplementary Table 1.** Cancer related AEs with positive signals for PPIs as a class.

| **Cancer site** | **PTs** | **N** | **PRR** | **χ^2^** |
| --- | --- | --- | --- | --- |
| Gastric | Adenocarcinoma gastric | 96 | 9.318 | 494.252 |
| Gastric | Gastric neoplasm | 61 | 4.662 | 141.596 |
| Gastric | Gastrinoma | 18 | 16.452 | 140.19 |
| Gastric | Gastrointestinal melanoma | 3 | 4.701 | 4.364 |
| Gastric | Gastrointestinal neoplasm | 48 | 2.314 | 31.175 |
| Gastric | Gastrooesophageal cancer | 5 | 2.964 | 4.067 |
| Gastric | Metastatic gastric cancer | 30 | 4.273 | 60.126 |
| Gastric | Gastrointestinal submucosal tumour | 4 | 14.624 | 22.515 |
| Gastric | Carcinoid tumour of the gastrointestinal tract | 9 | 3.037 | 9.014 |
| Gastric | Carcinoid tumour of the stomach | 37 | 11.273 | 221.765 |
| Intestinal | Adenocarcinoma of colon | 94 | 3.525 | 144.212 |
| Intestinal | Adenomatous polyposis coli | 14 | 6.268 | 44.018 |
| Intestinal | Carcinoid tumour of the small bowel | 13 | 5.381 | 33.645 |
| Intestinal | Colon cancer stage II | 15 | 3.104 | 16.866 |
| Intestinal | Duodenal neoplasm | 8 | 4.743 | 16.345 |
| Intestinal | Rectal adenocarcinoma | 18 | 2.256 | 10.257 |
| Intestinal | Rectal neoplasm | 16 | 3.618 | 23.727 |
| Pancreatic | Adenocarcinoma pancreas | 57 | 2.373 | 39.564 |
| Pancreatic | Ductal adenocarcinoma of pancreas | 8 | 3.026 | 7.764 |
| Pancreatic | Pancreatic carcinoma metastatic | 121 | 2.314 | 80.453 |
| Pancreatic | Pancreatic neuroendocrine tumour | 25 | 2.069 | 11.626 |
| Pancreatic | Intraductal papillary-mucinous carcinoma of pancreas | 4 | 3.656 | 4.45 |
| Hepatobiliary | Malignant neoplasm of ampulla of Vater | 9 | 2.323 | 4.943 |
| Hepatobiliary | Biliary neoplasm | 10 | 2.285 | 5.385 |
| Hepatobiliary | Cholangiocarcinoma | 54 | 2.318 | 35.372 |
| Hepatobiliary | Gallbladder adenocarcinoma | 13 | 12.399 | 79.591 |
| Oesophageal | Oesophageal cancer metastatic | 22 | 3.523 | 32.01 |
| Oesophageal | Oesophageal neoplasm | 22 | 5.08 | 55.124 |
| Oesophageal | Oesophageal squamous cell carcinoma | 15 | 2.675 | 12.507 |
| Oesophageal | Oesophageal squamous cell carcinoma metastatic | 5 | 15.669 | 31.607 |
| Oesophageal | Oesophageal adenocarcinoma | 36 | 4.412 | 76.141 |
| Abdominal wall and peritoneal | Abdominal wall neoplasm | 4 | 4.875 | 7.038 |
| Lip and oral cavity | Oropharyngeal neoplasm | 3 | 5.983 | 6.117 |
| Lip and oral cavity | Lip neoplasm malignant stage unspecified | 17 | 2.936 | 17.402 |
| Anal canal | Anal cancer stage 0 | 3 | 5.983 | 6.117 |
| Upper respiratory tract | Paranasal sinus neoplasm | 6 | 5.265 | 13.314 |
| Upper respiratory tract | Hypopharyngeal cancer | 6 | 3.656 | 7.686 |
| Upper respiratory tract | Laryngeal neoplasm | 22 | 4.685 | 49.425 |
| Lung | Adenosquamous cell lung cancer | 5 | 4.062 | 7.225 |
| Lung | Lung adenocarcinoma | 155 | 2.374 | 109.993 |
| Lung | Lung adenocarcinoma stage III | 14 | 11.812 | 82.862 |
| Lung | Lung neoplasm | 320 | 2.207 | 190.786 |
| Lung | Lung squamous cell carcinoma metastatic | 5 | 4.57 | 8.658 |
| Lung | Non-small cell lung cancer stage I | 4 | 5.162 | 7.628 |
| Lung | Non-small cell lung cancer stage II | 3 | 16.452 | 16.504 |
| Lung | Non-small cell lung cancer stage III | 3 | 5.062 | 4.869 |
| Lung | Non-small cell lung cancer stage IIIB | 8 | 9.75 | 37.386 |
| Lung | Small cell lung cancer metastatic | 23 | 3.386 | 31.377 |
| Lung | Squamous cell carcinoma of lung | 47 | 2.449 | 34.899 |
| Lung | Neuroendocrine tumour of the lung | 7 | 4.953 | 14.804 |
| Lung | Carcinoid tumour pulmonary | 13 | 2.144 | 6.182 |
| Bronchial | Metastatic bronchial carcinoma | 9 | 2.269 | 4.65 |
| Adrenal | Phaeochromocytoma malignant | 5 | 21.937 | 39.61 |
| Other and unspecified endocrine glands | Neuroendocrine carcinoma metastatic | 12 | 2.8 | 10.705 |
| Other and unspecified endocrine glands | Endocrine neoplasm malignant | 3 | 4.701 | 4.364 |
| Other and unspecified endocrine glands | Carcinoid tumour | 52 | 2.101 | 26.332 |
| Renal | Renal cell carcinoma stage IV | 13 | 4.527 | 26.631 |
| Renal | papillary renal cell carcinoma | 10 | 6.452 | 31.331 |
| Renal | Transitional cell cancer of the renal pelvis and ureter | 5 | 2.964 | 4.067 |
| Breast | Intraductal papillary breast neoplasm | 3 | 10.968 | 11.837 |
| Breast | Lobular breast carcinoma in situ | 8 | 3.734 | 11.351 |
| Breast | Phyllodes tumour | 7 | 3.839 | 10.108 |
| Penile | Penile squamous cell carcinoma | 4 | 4.178 | 5.572 |
| Ovarian and fallopian tube | Ovarian cancer stage I | 15 | 2.334 | 9.112 |
| Ovarian and fallopian tube | Ovarian epithelial cancer | 9 | 2.218 | 4.373 |
| Uterine and cervix | Cervix carcinoma | 21 | 6.876 | 75.672 |
| Vulvovaginal | Vaginal neoplasm | 10 | 3.596 | 13.855 |
| Haematologic | Bone marrow leukaemic cell infiltration | 5 | 4.062 | 7.225 |
| Haematologic | Refractory cytopenia with unilineage dysplasia | 24 | 2.041 | 10.678 |
| Lymphomas | Anaplastic large cell lymphoma T- and null-cell types | 13 | 2.417 | 8.437 |
| Lymphomas | Anaplastic large cell lymphoma T- and null-cell types stage II | 3 | 21.937 | 20.027 |
| Lymphomas | B-cell lymphoma refractory | 6 | 3.375 | 6.671 |
| Lymphomas | Cutaneous T-cell lymphoma stage III | 3 | 7.312 | 7.81 |
| Lymphomas | Diffuse large B-cell lymphoma stage IV | 19 | 3.969 | 33.159 |
| Lymphomas | High grade B-cell lymphoma Burkitt-like lymphoma | 4 | 43.873 | 41.918 |
| Lymphomas | high-grade B-cell lymphoma | 8 | 3.25 | 8.901 |
| Lymphomas | Nodal marginal zone B-cell lymphoma stage IV | 4 | 87.746 | 51.665 |
| Lymphomas | Non-Hodgkin's lymphoma refractory | 9 | 12.339 | 52.673 |
| Lymphomas | Hodgkin's disease stage II | 8 | 6.75 | 25.543 |
| Nervous system | Brain stem glioma | 30 | 10.968 | 174.309 |
| Nervous system | Glioblastoma multiforme | 43 | 2.106 | 21.733 |
| Head and neck | Retro-orbital neoplasm | 10 | 10.446 | 51.366 |
| Head and neck | Ear neoplasm | 13 | 2.228 | 6.864 |
| Head and neck | Ear neoplasm malignant | 12 | 2.925 | 11.696 |
| Head and neck | external ear neoplasm malignant | 3 | 8.226 | 8.9 |
| Skin | Basosquamous carcinoma of skin | 4 | 4.178 | 5.572 |
| Skin | Nodular melanoma | 8 | 3.134 | 8.312 |
| Bone | Bone neoplasm | 50 | 2.069 | 24.242 |
| Bone | Intraosseous angioma | 3 | 7.312 | 7.81 |
| Soft tissue | Dermatofibrosarcoma protuberans | 10 | 4.219 | 17.87 |
| Soft tissue | Neurofibrosarcoma | 12 | 4.387 | 23.284 |
| Soft tissue | Spindle cell sarcoma | 10 | 3.134 | 10.836 |
| Mediastinal | Malignant mediastinal neoplasm | 34 | 49.723 | 481.447 |
| Mediastinal | Malignant neoplasm of thymus | 9 | 12.339 | 52.673 |
| Site unspecified | malignant polyp | 15 | 5.983 | 44.902 |
| Site unspecified | Mucoepidermoid carcinoma | 17 | 6.215 | 53.8 |

AEs, adverse events; PPIs, proton pump inhibitors; PTs, Preferred Terms; PRR, proportional reporting ratio; χ^2^, chi-square.
