# Supplementary material for: Cancer related adverse events associated with use of proton pump inhibitors and histamine-2 receptor antagonists: A real-world analysis using the FDA adverse event reporting system
Source: PLoS One. 2025 Aug 12;20(8):e0329385. doi: 10.1371/journal.pone.0329385 (PMC12342331; doi:10.1371/journal.pone.0329385)
Supplement: S2 Table — (DOCX) [file pone.0329385.s002.docx]

**Supplementary Table 2.** Cancer related AEs with positive signals for omeprazole.

| **Cancer site** | **PTs** | **N** | **PRR** | **χ^2^** |
| --- | --- | --- | --- | --- |
| Gastric | Adenocarcinoma gastric | 59 | 12.1 | 481.523 |
| Gastric | Carcinoid tumour of the gastrointestinal tract | 7 | 5.635 | 20.079 |
| Gastric | Carcinoid tumour of the stomach | 13 | 7.304 | 56.77 |
| Gastric | Gastric neoplasm | 29 | 4.903 | 79.002 |
| Gastric | Gastrinoma | 7 | 10.788 | 43.827 |
| Gastric | Gastrointestinal lymphoma | 6 | 3.336 | 7.139 |
| Gastric | Gastrointestinal neoplasm | 19 | 2.117 | 9.714 |
| Gastric | Metastatic gastric cancer | 20 | 6.578 | 79.327 |
| Intestinal | Adenocarcinoma of colon | 33 | 2.755 | 33.431 |
| Intestinal | Adenomatous polyposis coli | 7 | 6.742 | 25.453 |
| Intestinal | Mesenteric neoplasm | 4 | 3.657 | 4.919 |
| Pancreatic | Adenocarcinoma pancreas | 28 | 2.716 | 27.269 |
| Pancreatic | Ductal adenocarcinoma of pancreas | 4 | 3.48 | 4.48 |
| Pancreatic | Pancreatic carcinoma metastatic | 53 | 2.353 | 38.198 |
| Hepatobiliary | Hepatic neoplasm | 73 | 2.109 | 39.894 |
| Hepatobiliary | Cholangiocarcinoma | 23 | 2.289 | 14.779 |
| Hepatobiliary | Gallbladder adenocarcinoma | 12 | 26.969 | 182.803 |
| Oesophageal | Oesophageal adenocarcinoma | 21 | 5.839 | 71.602 |
| Oesophageal | Oesophageal cancer metastatic | 14 | 5.208 | 39.586 |
| Abdominal wall and peritoneal | Abdominal wall neoplasm | 3 | 8.516 | 11.212 |
| Lip and oral cavity | Lip neoplasm malignant stage unspecified | 7 | 2.756 | 5.847 |
| Anal canal | Anal cancer stage 0 | 3 | 14.71 | 20.147 |
| Upper respiratory tract | Laryngeal neoplasm | 6 | 2.72 | 4.655 |
| Upper respiratory tract | Paranasal sinus neoplasm | 5 | 10.373 | 27.96 |
| Upper respiratory tract | Tonsil cancer | 22 | 2.313 | 14.47 |
| Lung | Adenosquamous cell lung cancer | 5 | 9.988 | 26.836 |
| Lung | Lung adenocarcinoma stage III | 3 | 4.373 | 4.392 |
| Lung | Lung adenocarcinoma stage IV | 10 | 4.35 | 20.819 |
| Lung | Lung neoplasm | 132 | 2.113 | 73.436 |
| Lung | Non-small cell lung cancer stage II | 3 | 40.453 | 44.998 |
| Lung | Non-small cell lung cancer stage IIIB | 4 | 9.807 | 19.716 |
| Renal | papillary renal cell carcinoma | 5 | 6.915 | 17.402 |
| Renal | Renal cell carcinoma stage IV | 9 | 7.245 | 37.289 |
| Urinary tract | Transitional cell carcinoma | 20 | 2.032 | 9.109 |
| Breast | Hormone receptor positive breast cancer | 8 | 3.294 | 9.943 |
| Breast | Intraductal papillary breast neoplasm | 3 | 26.969 | 33.933 |
| Prostatic | Neoplasm prostate | 8 | 2.345 | 4.675 |
| Testicular | Testicular neoplasm | 5 | 4.731 | 10.259 |
| Uterine and cervix | Papillary serous endometrial carcinoma | 3 | 8.091 | 10.539 |
| Haematologic | Myeloproliferative neoplasm | 16 | 2.08 | 7.608 |
| Lymphomas | Diffuse large B-cell lymphoma refractory | 14 | 3.371 | 19.761 |
| Lymphomas | Diffuse large B-cell lymphoma stage IV | 10 | 4.731 | 23.673 |
| Lymphomas | Extranodal marginal zone B-cell lymphoma (MALT type) | 12 | 2.209 | 6.491 |
| Lymphomas | Follicular lymphoma | 7 | 4.29 | 13.406 |
| Lymphomas | High grade B-cell lymphoma Burkitt-like lymphoma | 3 | 53.938 | 53.306 |
| Lymphomas | High-grade B-cell lymphoma | 8 | 7.991 | 36.638 |
| Lymphomas | Nodal marginal zone B-cell lymphoma stage IV | 4 | 215.751 | 130.056 |
| Lymphomas | Non-Hodgkin's lymphoma refractory | 9 | 30.34 | 144.862 |
| Nervous system | Ependymoma | 4 | 3.596 | 4.767 |
| Head and neck | Ear neoplasm | 7 | 2.818 | 6.14 |
| Soft tissue | Inflammatory myofibroblastic tumour | 5 | 4.086 | 8.109 |
| Soft tissue | Myxofibrosarcoma | 3 | 7.705 | 9.924 |
| Soft tissue | Spindle cell sarcoma | 7 | 5.172 | 17.794 |
| Mediastinal | Malignant mediastinal neoplasm | 33 | 111.246 | 1140.907 |
| Site unspecified | Adenocarcinoma metastatic | 3 | 8.516 | 11.212 |
| Site unspecified | Squamous cell carcinoma of head and neck | 5 | 3.03 | 4.63 |

AEs, adverse events; PTs, Preferred Terms; PRR, proportional reporting ratio; χ^2^, chi-square.
