# Supplementary material for: Cancer related adverse events associated with use of proton pump inhibitors and histamine-2 receptor antagonists: A real-world analysis using the FDA adverse event reporting system
Source: PLoS One. 2025 Aug 12;20(8):e0329385. doi: 10.1371/journal.pone.0329385 (PMC12342331; doi:10.1371/journal.pone.0329385)
Supplement: S3 Table — (DOCX) [file pone.0329385.s003.docx]

**Supplementary Table 3.** Cancer related AEs with positive signals for lansoprazole.

| Cancer site | PTs | N | PRR | χ^2^ |
| --- | --- | --- | --- | --- |
| Gastric | Adenocarcinoma gastric | 36 | 13.165 | 348.605 |
| Gastric | Carcinoid tumour of the stomach | 18 | 20.687 | 265.204 |
| Gastric | Gastric neoplasm | 9 | 2.777 | 8.296 |
| Gastric | Gastrinoma | 5 | 14.133 | 42.71 |
| Gastric | Gastrooesophageal cancer | 3 | 8.045 | 11.216 |
| Gastric | Metastatic gastric cancer | 10 | 6.011 | 34.862 |
| Intestinal | Adenocarcinoma of colon | 21 | 3.338 | 31.077 |
| Hepatobiliary | Hepatic cancer metastatic | 15 | 3.826 | 27.521 |
| Oesophageal | Oesophageal adenocarcinoma | 7 | 3.52 | 9.879 |
| Oesophageal | Oesophageal squamous cell carcinoma | 5 | 3.932 | 7.875 |
| Lip and oral cavity | Oropharyngeal neoplasm | 3 | 28.523 | 42.673 |
| Upper respiratory tract | Pharyngeal neoplasm | 3 | 4.24 | 4.341 |
| Lung | Lung adenocarcinoma | 32 | 2.152 | 18.222 |
| Lung | Small cell lung cancer metastatic | 7 | 4.437 | 14.705 |
| Lung | Squamous cell carcinoma of lung | 15 | 3.463 | 23.087 |
| Thyroid | Follicular thyroid cancer | 3 | 4.683 | 5.139 |
| Other and unspecified endocrine glands | Neuroendocrine carcinoma metastatic | 4 | 4.101 | 6.265 |
| Other and unspecified endocrine glands | Neuroendocrine tumour | 26 | 3.357 | 39.42 |
| Renal | Clear cell renal cell carcinoma | 7 | 2.838 | 6.405 |
| Renal | papillary renal cell carcinoma | 9 | 26.894 | 158.293 |
| Ureteric | Ureteral neoplasm | 3 | 9.508 | 13.803 |
| Breast | Invasive lobular breast carcinoma | 9 | 5.034 | 24.005 |
| Breast | Lobular breast carcinoma in situ | 4 | 8.203 | 17.201 |
| Breast | Phyllodes tumour | 5 | 12.451 | 37.29 |
| Uterine and cervix | Endometrial adenocarcinoma | 7 | 3.013 | 7.281 |
| Uterine and cervix | Cervix carcinoma stage IV | 3 | 4.754 | 5.267 |
| Haematologic | Marrow hyperplasia | 11 | 3.214 | 14.177 |
| Lymphomas | Anaplastic large cell lymphoma T- and null-cell types | 6 | 5.02 | 14.762 |
| Lymphomas | Anaplastic large cell lymphoma T- and null-cell types stage II | 3 | 104.586 | 106.047 |
| Lymphomas | Diffuse large B-cell lymphoma stage IV | 4 | 3.486 | 4.649 |
| Lymphomas | Follicular lymphoma | 6 | 7.051 | 23.746 |
| Lymphomas | Nodal marginal zone B-cell lymphoma stage IV | 4 | 418.344 | 254.14 |
| Lymphomas | Hodgkin's disease stage II | 7 | 27.115 | 119.662 |
| Nervous system | Brain stem glioma | 28 | 47.232 | 841.04 |
| Nervous system | Ganglioglioma | 3 | 16.514 | 25.445 |
| Nervous system | Meningeal neoplasm | 3 | 8.965 | 12.848 |
| Bone | Bone neoplasm | 16 | 2.967 | 18.405 |
| Bone | Intraosseous angioma | 3 | 34.862 | 50.585 |
| Soft tissue | Spindle cell sarcoma | 3 | 4.075 | 4.045 |
| Mediastinal | Malignant neoplasm of thymus | 7 | 40.672 | 167.261 |
| Site unspecified | Abdominal neoplasm | 13 | 2.541 | 10.395 |
| Site unspecified | Malignant neoplasm of unknown primary site | 5 | 3.208 | 5.369 |

AEs, adverse events; PTs, Preferred Terms; PRR, proportional reporting ratio; χ^2^, chi-square.
