# Supplementary material for: Cancer related adverse events associated with use of proton pump inhibitors and histamine-2 receptor antagonists: A real-world analysis using the FDA adverse event reporting system
Source: PLoS One. 2025 Aug 12;20(8):e0329385. doi: 10.1371/journal.pone.0329385 (PMC12342331; doi:10.1371/journal.pone.0329385)
Supplement: S4 Table — (DOCX) [file pone.0329385.s004.docx]

**Supplementary Table 4.** Cancer related AEs with positive signals for rabeprazole.

| **Cancer site** | **PTs** | **N** | **PRR** | **χ^2^** |
| --- | --- | --- | --- | --- |
| Gastric | Adenocarcinoma gastric | 8 | 12.476 | 71.522 |
| Gastric | Gastrointestinal neoplasm | 7 | 6.911 | 29.302 |
| Gastric | Metastatic gastric cancer | 3 | 8.116 | 12.067 |
| Intestinal | Adenocarcinoma of colon | 7 | 5.101 | 18.956 |
| Pancreatic | Pancreatic neuroendocrine tumour | 5 | 8.591 | 25.908 |
| Hepatobiliary | Bile duct cancer | 5 | 3.001 | 4.786 |
| Lip and oral cavity | Lip and/or oral cavity cancer | 8 | 4.524 | 18.4 |
| Upper respiratory tract | Pharyngeal neoplasm | 4 | 26.832 | 71.366 |
| Lung | Lung adenocarcinoma | 8 | 2.481 | 5.639 |
| Lung | Non-small cell lung cancer stage IIIB | 3 | 63.872 | 113.237 |
| Renal | Renal cell carcinoma | 16 | 2.203 | 9.305 |
| Ureteric | Ureteric cancer | 3 | 6.278 | 8.44 |
| Haematologic | Bone marrow leukaemic cell infiltration | 5 | 90.683 | 302.194 |
| Haematologic | Marrow hyperplasia | 3 | 4.014 | 4.072 |
| Haematologic | Transformation to acute myeloid leukaemia | 3 | 5.209 | 6.355 |
| Haematologic | Chronic myeloid leukaemia transformation | 5 | 8.807 | 26.743 |
| Lymphomas | Epstein-Barr virus associated lymphoma | 3 | 9.125 | 14.063 |
| Lymphomas | Adult T-cell lymphoma/leukaemia | 4 | 6.996 | 14.774 |
| Lymphomas | Anaplastic large cell lymphoma T- and null-cell types | 7 | 27.644 | 145.821 |
| Lymphomas | Peripheral T-cell lymphoma unspecified | 4 | 7.309 | 15.686 |
| Lymphomas | Hodgkin's disease stage IV | 3 | 35.831 | 64.922 |
| Lymphomas | Lymphoma | 37 | 2.012 | 17.781 |
| Nervous system | Schwannoma | 3 | 10.062 | 15.919 |
| Skin | Squamous cell carcinoma of skin | 20 | 3.978 | 41.326 |
| Bone | Bone neoplasm | 8 | 6.849 | 33.841 |
| Soft tissue | Liposarcoma | 3 | 8.253 | 12.338 |
| Site unspecified | Malignant polyp | 15 | 133.551 | 1447.912 |
| Site unspecified | Second primary malignancy | 35 | 3.845 | 70.343 |

AEs, adverse events; PTs, Preferred Terms; PRR, proportional reporting ratio; χ^2^, chi-square.
