# Supplementary material for: Cancer related adverse events associated with use of proton pump inhibitors and histamine-2 receptor antagonists: A real-world analysis using the FDA adverse event reporting system
Source: PLoS One. 2025 Aug 12;20(8):e0329385. doi: 10.1371/journal.pone.0329385 (PMC12342331; doi:10.1371/journal.pone.0329385)
Supplement: S5 Table — (DOCX) [file pone.0329385.s005.docx]

**Supplementary Table 5.** Cancer related AEs with positive signals for pantoprazole.

| **Cancer site** | **PTs** | **N** | **PRR** | **χ^2^** |
| --- | --- | --- | --- | --- |
| Gastric | Adenocarcinoma gastric | 59 | 17.783 | 749.492 |
| Gastric | Carcinoid tumour of the stomach | 18 | 15.68 | 194.311 |
| Gastric | Gastric neoplasm | 16 | 3.82 | 29.116 |
| Gastric | Gastrinoma | 4 | 8.344 | 17.149 |
| Gastric | Gastrointestinal cancer metastatic | 6 | 5.662 | 17.317 |
| Gastric | Metastatic gastric cancer | 11 | 5.04 | 29.76 |
| Intestinal | Adenocarcinoma of colon | 28 | 3.409 | 43.405 |
| Intestinal | Adenomatous polyposis coli | 6 | 8.344 | 28.685 |
| Intestinal | Carcinoid tumour of the small bowel | 10 | 14.155 | 92.742 |
| Intestinal | Colon cancer metastatic | 23 | 2.001 | 10.273 |
| Intestinal | Intestinal adenocarcinoma | 4 | 3.373 | 4.308 |
| Intestinal | Rectal adenocarcinoma | 9 | 3.877 | 15.649 |
| Pancreatic | Adenocarcinoma pancreas | 16 | 2.233 | 9.415 |
| Pancreatic | Ductal adenocarcinoma of pancreas | 4 | 5.114 | 8.831 |
| Pancreatic | Pancreatic carcinoma metastatic | 37 | 2.383 | 27.48 |
| Hepatobiliary | Malignant neoplasm of ampulla of Vater | 4 | 3.523 | 4.69 |
| Hepatobiliary | Cholangiocarcinoma | 14 | 2.014 | 6.006 |
| Oesophageal | Oesophageal cancer metastatic | 6 | 3.109 | 6.331 |
| Oesophageal | Oesophageal neoplasm | 5 | 3.539 | 6.431 |
| Oesophageal | Oesophageal squamous cell carcinoma metastatic | 3 | 26.423 | 37.423 |
| Abdominal wall and peritoneal | Peritoneal neoplasm | 3 | 5.17 | 5.923 |
| Lip and oral cavity | Squamous cell carcinoma of the tongue | 7 | 3.801 | 11.212 |
| Lung | Carcinoid tumour pulmonary | 9 | 5.208 | 24.852 |
| Lung | Lung adenocarcinoma stage III | 10 | 26.423 | 164.66 |
| Lung | Lung squamous cell carcinoma stage I | 3 | 6.098 | 7.562 |
| Lung | Neuroendocrine tumour of the lung | 7 | 17.9 | 77.69 |
| Lung | Small cell lung cancer | 26 | 2.287 | 17.069 |
| Lung | Small cell lung cancer metastatic | 7 | 3.363 | 8.972 |
| Bronchial | Bronchial neoplasm | 4 | 7.734 | 15.606 |
| Bronchial | Metastatic bronchial carcinoma | 6 | 5.285 | 15.685 |
| Other and unspecified endocrine glands | Endocrine neoplasm malignant | 3 | 16.987 | 25.035 |
| Other and unspecified endocrine glands | Carcinoid tumour | 24 | 3.332 | 35.358 |
| Bladder | Bladder transitional cell carcinoma | 26 | 2.626 | 23.761 |
| Urinary tract | Transitional cell carcinoma | 19 | 2.831 | 19.974 |
| Breast | Breast neoplasm | 19 | 2.445 | 14.354 |
| Prostatic | Neoplasm prostate | 11 | 4.818 | 27.832 |
| Penile | Penile squamous cell carcinoma | 3 | 10.81 | 15.573 |
| Ovarian and fallopian tube | Ovarian cancer stage I | 12 | 6.606 | 47.587 |
| Ovarian and fallopian tube | Ovarian epithelial cancer | 4 | 3.373 | 4.308 |
| Lymphomas | B-cell lymphoma stage I | 3 | 9.909 | 14.093 |
| Lymphomas | Cutaneous T-cell lymphoma stage III | 3 | 26.423 | 37.423 |
| Lymphomas | Epstein-Barr virus associated lymphoma | 7 | 3.534 | 9.845 |
| Lymphomas | Follicle centre lymphoma, follicular grade I, II, III | 3 | 6.098 | 7.562 |
| Lymphomas | Non-Hodgkin's lymphoma stage I | 4 | 9.326 | 19.594 |
| Lymphomas | Hodgkin's disease stage IV | 3 | 5.8 | 7.038 |
| Lymphomas | Plasmacytoma | 24 | 2.116 | 12.685 |
| Nervous system | Glioblastoma multiforme | 23 | 3.896 | 44.436 |
| Head and neck | Retro-orbital neoplasm | 10 | 37.748 | 217.79 |
| Head and neck | Ear neoplasm malignant | 8 | 6.746 | 30.923 |
| Skin | Malignant melanoma in situ | 26 | 2.445 | 20.143 |
| Skin | Metastatic malignant melanoma | 36 | 2.205 | 21.905 |
| Skin | Nodular melanoma | 8 | 11.324 | 57.059 |
| Skin | Sebaceous carcinoma | 3 | 4.756 | 5.191 |
| Bone | Bone neoplasm | 22 | 3.125 | 28.557 |
| Soft tissue | Rhabdomyosarcoma | 4 | 3.82 | 5.454 |
| Soft tissue | Neurofibrosarcoma | 12 | 15.854 | 126.92 |
| Site unspecified | Adenocarcinoma | 35 | 2.263 | 22.762 |
| Site unspecified | Mucoepidermoid carcinoma | 12 | 14.635 | 117.288 |
| Site unspecified | Signet-ring cell carcinoma | 3 | 7.206 | 9.503 |

AEs, adverse events; PTs, Preferred Terms; PRR, proportional reporting ratio; χ^2^, chi-square.
