# Supplementary material for: Cancer related adverse events associated with use of proton pump inhibitors and histamine-2 receptor antagonists: A real-world analysis using the FDA adverse event reporting system
Source: PLoS One. 2025 Aug 12;20(8):e0329385. doi: 10.1371/journal.pone.0329385 (PMC12342331; doi:10.1371/journal.pone.0329385)
Supplement: S6 Table — (DOCX) [file pone.0329385.s006.docx]

**Supplementary Table 6.** Cancer related AEs with positive signals for esomeprazole.

| **Cancer site** | **PTs** | **N** | **PRR** | **χ^2^** |
| --- | --- | --- | --- | --- |
| Gastric | Adenocarcinoma gastric | 35 | 8.984 | 214.199 |
| Gastric | Carcinoid tumour of the stomach | 10 | 7.441 | 44.887 |
| Gastric | Gastric neoplasm | 21 | 4.731 | 54.565 |
| Gastric | Gastrinoma | 11 | 26.141 | 177.955 |
| Gastric | Gastrointestinal neoplasm | 18 | 2.734 | 17.433 |
| Gastric | Metastatic gastric cancer | 24 | 11.05 | 182.016 |
| Gastric | Gastrointestinal submucosal tumour | 3 | 31.573 | 42.37 |
| Intestinal | Colon cancer stage II | 6 | 3.844 | 9.414 |
| Intestinal | Rectal neoplasm | 12 | 8.753 | 66.799 |
| Pancreatic | Pancreatic neuroendocrine tumour | 11 | 2.905 | 11.424 |
| Oesophageal | Oesophageal adenocarcinoma | 11 | 3.972 | 20.444 |
| Oesophageal | Oesophageal neoplasm | 12 | 8.419 | 63.825 |
| Lip and oral cavity | Lip neoplasm malignant stage unspecified | 9 | 4.911 | 22.697 |
| Upper respiratory tract | Hypopharyngeal cancer | 5 | 9.955 | 27.938 |
| Upper respiratory tract | Laryngeal neoplasm | 14 | 9.292 | 84.678 |
| Upper respiratory tract | Throat cancer | 107 | 3.014 | 136.454 |
| Lung | Lung adenocarcinoma stage III | 7 | 15.627 | 67.307 |
| Lung | Lung neoplasm | 103 | 2.233 | 66.881 |
| Lung | Squamous cell carcinoma of lung | 15 | 2.439 | 10.96 |
| Bronchial | Bronchial neoplasm | 5 | 9.209 | 25.546 |
| Adrenal | Phaeochromocytoma malignant | 5 | 73.67 | 144.273 |
| Renal | Renal cell carcinoma stage IV | 4 | 4.093 | 6.136 |
| Ureteric | Ureteral neoplasm | 3 | 6.697 | 8.56 |
| Ovarian and fallopian tube | Ovarian cancer stage I | 14 | 7.263 | 63.171 |
| Uterine and cervix | Endometrial cancer stage II | 4 | 9.506 | 19.869 |
| Uterine and cervix | Endometrial neoplasm | 3 | 7.893 | 10.612 |
| Vulvovaginal | Vaginal neoplasm | 10 | 12.077 | 77.91 |
| Lymphomas | B-cell lymphoma refractory | 5 | 9.209 | 25.546 |
| Lymphomas | Follicular lymphoma | 5 | 4.093 | 8.3 |
| Lymphomas | Hepatosplenic T-cell lymphoma | 11 | 2.517 | 8.293 |
| Lymphomas | Nodal marginal zone B-cell lymphoma stage IV | 4 | 294.68 | 178.397 |
| Lymphomas | Metastatic lymphoma | 7 | 2.503 | 4.727 |
| Head and neck | Ocular neoplasm | 10 | 3.044 | 11.268 |
| Head and neck | Optic nerve neoplasm | 3 | 4.911 | 5.438 |
| Bone | Bone neoplasm | 24 | 3.18 | 32.299 |
| Soft tissue | Dermatofibrosarcoma protuberans | 7 | 9.376 | 39.24 |
| Site unspecified | Head and neck cancer | 12 | 2.13 | 5.925 |
| Site unspecified | Mucoepidermoid carcinoma | 12 | 13.601 | 107.723 |

AEs, adverse events; PTs, Preferred Terms; PRR, proportional reporting ratio; χ^2^, chi-square.
