# Supplementary material for: Cancer related adverse events associated with use of proton pump inhibitors and histamine-2 receptor antagonists: A real-world analysis using the FDA adverse event reporting system
Source: PLoS One. 2025 Aug 12;20(8):e0329385. doi: 10.1371/journal.pone.0329385 (PMC12342331; doi:10.1371/journal.pone.0329385)
Supplement: S7 Table — (DOCX) [file pone.0329385.s007.docx]

**Supplementary Table 7.** Cancer related AEs with positive signals for dexlansoprazole.

| **Cancer site** | **PTs** | **N** | **PRR** | **χ^2^** |
| --- | --- | --- | --- | --- |
| Gastric | Adenocarcinoma gastric | 7 | 7.85 | 34.492 |
| Gastric | Metastatic gastric cancer | 5 | 9.868 | 30.595 |
| Upper respiratory tract | Laryngeal neoplasm | 3 | 8.687 | 13.104 |
| Thyroid | Papillary thyroid cancer | 6 | 3.207 | 6.97 |
| Other and unspecified endocrine glands | Carcinoid tumour | 7 | 4.206 | 13.876 |
| Uterine and cervix | Cervix neoplasm | 21 | 110.727 | 1655.786 |

AEs, adverse events; PTs, Preferred Terms; PRR, proportional reporting ratio; χ^2^, chi-square.
