# Supplementary material for: Cancer related adverse events associated with use of proton pump inhibitors and histamine-2 receptor antagonists: A real-world analysis using the FDA adverse event reporting system
Source: PLoS One. 2025 Aug 12;20(8):e0329385. doi: 10.1371/journal.pone.0329385 (PMC12342331; doi:10.1371/journal.pone.0329385)
Supplement: S8 Table — (DOCX) [file pone.0329385.s008.docx]

**Supplementary Table 8.** Cancer related AEs with positive signals for ranitidine.

| **Cancer site** | **PTs** | **N** | **PRR** | **χ^2^** |
| --- | --- | --- | --- | --- |
| Gastric | Gastric cancer | 14863 | 112.184 | 347036.03 |
| Gastric | Gastric cancer stage 0 | 46 | 692.321 | 1293.528 |
| Gastric | Gastrooesophageal cancer | 7 | 6.02 | 20.289 |
| Gastric | Linitis plastica | 3 | 22.576 | 23.758 |
| Gastric | Gastrointestinal carcinoma | 5242 | 110.807 | 121899.91 |
| Gastric | Gastrointestinal stromal tumour | 154 | 3.09 | 195.548 |
| Gastric | Gastrointestinal cancer metastatic | 11 | 4.191 | 20.657 |
| Gastric | Carcinoid tumour of the stomach | 12 | 3.724 | 18.846 |
| Gastric | Carcinoid tumour of the gastrointestinal tract | 8 | 3.649 | 11.386 |
| Intestinal | Small intestine carcinoma | 1325 | 95.874 | 29705.01 |
| Intestinal | Colorectal cancer | 40027 | 1005.717 | >10^6^ |
| Intestinal | Colon cancer | 1502 | 6.227 | 5460.806 |
| Intestinal | Rectal cancer | 187 | 3.832 | 344.611 |
| Intestinal | Colorectal carcinoma stage 0 | 18 | 541.816 | 482.42 |
| Intestinal | Colon cancer stage 0 | 7 | 11.09 | 39.65 |
| Intestinal | Mucinous adenocarcinoma of appendix | 6 | 12.9 | 37.902 |
| Intestinal | Adenomatous polyposis coli | 6 | 3.169 | 6.157 |
| Intestinal | Malignant mesenteric neoplasm | 6 | 6.689 | 19.187 |
| Intestinal | Mesenteric neoplasm | 13 | 7.826 | 55.96 |
| Intestinal | Neoplasm of appendix | 17 | 8.823 | 85.043 |
| Intestinal | Appendix cancer | 585 | 181.536 | 14912.945 |
| Intestinal | Carcinoid tumour of the small bowel | 7 | 3.571 | 9.332 |
| Pancreatic | Pancreatic carcinoma | 12948 | 50.689 | 235390.47 |
| Hepatobiliary | Hepatic cancer | 14604 | 86.077 | 318706.38 |
| Hepatobiliary | Hepatic cancer metastatic | 28 | 2.123 | 14.472 |
| Hepatobiliary | Bile duct cancer | 126 | 5.457 | 384.432 |
| Hepatobiliary | Cholangiocarcinoma | 75 | 4.607 | 180.497 |
| Hepatobiliary | Bile duct adenocarcinoma | 14 | 22.18 | 150.668 |
| Hepatobiliary | Gallbladder cancer | 740 | 58.772 | 14214.531 |
| Hepatobiliary | Carcinoid tumour of the liver | 8 | 26.756 | 91.393 |
| Oesophageal | Oesophageal carcinoma | 19802 | 284.65 | 536283.75 |
| Oesophageal | Oesophageal cancer metastatic | 21 | 4.581 | 47.854 |
| Oesophageal | Oesophageal carcinoma stage 0 | 12 | 180.605 | 280.254 |
| Abdominal wall and peritoneal | Malignant peritoneal neoplasm | 33 | 3.763 | 56.991 |
| Abdominal wall and peritoneal | Abdominal wall neoplasm | 3 | 4.753 | 4.694 |
| Lip and oral cavity | Lip and/or oral cavity cancer | 191 | 8.418 | 969.727 |
| Lip and oral cavity | Tongue cancer metastatic | 7 | 6.385 | 21.839 |
| Lip and oral cavity | Malignant palate neoplasm | 6 | 5.826 | 16.136 |
| Lip and oral cavity | Tongue carcinoma stage 0 | 3 | 15.05 | 17.448 |
| Lip and oral cavity | Tongue neoplasm | 18 | 4.369 | 37.859 |
| Lip and oral cavity | Tongue neoplasm malignant stage unspecified | 474 | 22.54 | 5567.027 |
| Lip and oral cavity | Salivary gland cancer | 54 | 6.83 | 214.137 |
| Anal canal | Anal cancer | 158 | 12.385 | 1163.311 |
| Anal canal | Anal squamous cell carcinoma | 8 | 2.867 | 7.205 |
| Upper respiratory tract | Throat cancer | 1500 | 36.949 | 23541.756 |
| Upper respiratory tract | Pharyngeal cancer | 8 | 2.676 | 6.202 |
| Upper respiratory tract | Hypopharyngeal cancer | 6 | 5.017 | 13.174 |
| Upper respiratory tract | Nasopharyngeal cancer | 114 | 27.452 | 1505.479 |
| Upper respiratory tract | Oropharyngeal cancer | 26 | 5.974 | 85.613 |
| Upper respiratory tract | Oropharyngeal squamous cell carcinoma | 5 | 3.272 | 5.154 |
| Upper respiratory tract | Tonsil cancer | 170 | 14.02 | 1393.224 |
| Upper respiratory tract | Laryngeal cancer | 234 | 14.891 | 2019.21 |
| Upper respiratory tract | Epiglottic cancer | 17 | 17.057 | 153.604 |
| Upper respiratory tract | Laryngeal squamous cell carcinoma | 12 | 9.506 | 62.892 |
| Lung | Lung neoplasm malignant | 13542 | 28.176 | 183781.77 |
| Lung | Lung carcinoma cell type unspecified stage 0 | 22 | 21.362 | 237.597 |
| Thyroid | Thyroid cancer | 5109 | 43.553 | 86856.75 |
| Thyroid | Thyroid cancer stage 0 | 49 | 1474.944 | 1413.194 |
| Adrenal | Adrenal gland cancer | 125 | 24.433 | 1537.292 |
| Adrenal | Adrenal gland cancer metastatic | 3 | 5.644 | 6.036 |
| Other and unspecified endocrine glands | Neuroendocrine carcinoma | 116 | 12.515 | 859.737 |
| Other and unspecified endocrine glands | Paraganglion neoplasm | 13 | 5.671 | 38.125 |
| Other and unspecified endocrine glands | Endocrine neoplasm malignant | 4 | 9.262 | 16.488 |
| Other and unspecified endocrine glands | Carcinoid tumour | 87 | 5.155 | 245.126 |
| Renal | Renal cancer | 36457 | 284.666 | 988981.9 |
| Renal | Clear cell renal cell carcinoma | 32 | 4.134 | 64.033 |
| Renal | Papillary renal cell carcinoma | 6 | 4.753 | 12.189 |
| Renal | Malignant neoplasm of renal pelvis | 6 | 2.779 | 4.684 |
| Renal | Transitional cell cancer of the renal pelvis and ureter | 11 | 10.681 | 64.05 |
| Ureteric | Ureteric cancer | 67 | 11.863 | 470.064 |
| Ureteric | Ureteral neoplasm | 6 | 6.02 | 16.832 |
| Bladder | Bladder cancer | 32470 | 92.73 | 724765 |
| Bladder | Bladder cancer recurrent | 32 | 2.261 | 19.741 |
| Urinary tract | Urethral cancer | 75 | 49.078 | 1324.071 |
| Urinary tract | Urinary tract neoplasm | 5 | 6.02 | 13.388 |
| Urinary tract | Transitional cell carcinoma | 42 | 2.484 | 32.99 |
| Breast | Breast cancer | 44155 | 32.144 | 649211.06 |
| Breast | Breast cancer female | 1988 | 4.019 | 3979.908 |
| Breast | Intraductal proliferative breast lesion | 69 | 5.041 | 187.911 |
| Breast | Breast cancer male | 65 | 9.186 | 356.844 |
| Breast | Invasive lobular breast carcinoma | 30 | 5.44 | 88.23 |
| Breast | Lobular breast carcinoma in situ | 11 | 7.525 | 44.544 |
| Prostatic | Prostate cancer | 50069 | 151.029 | >10^6^ |
| Prostatic | Prostate cancer stage 0 | 203 | 763.81 | 5833.686 |
| Prostatic | Prostate cancer recurrent | 25 | 3.782 | 42.924 |
| Testicular | Testis cancer | 2688 | 154.706 | 66852.89 |
| Testicular | Seminoma | 12 | 2.301 | 6.997 |
| Testicular | Testicular cancer metastatic | 6 | 3.926 | 9.056 |
| Penile | Penile cancer | 21 | 5.963 | 68.191 |
| Ovarian and fallopian tube | Ovarian cancer | 2534 | 20.157 | 27635.889 |
| Ovarian and fallopian tube | Fallopian tube cancer | 9 | 6.02 | 27.224 |
| Ovarian and fallopian tube | Brenner tumour | 3 | 18.061 | 20.204 |
| Uterine and cervix | Endometrial cancer | 138 | 3.476 | 215.94 |
| Uterine and cervix | Uterine cancer | 4209 | 70.621 | 86358.305 |
| Uterine and cervix | Cervix carcinoma | 1259 | 26.839 | 16546.582 |
| Uterine and cervix | Cervix carcinoma stage 0 | 34 | 6.603 | 127.862 |
| Vulvovaginal | Vaginal cancer | 37 | 7.681 | 165.847 |
| Vulvovaginal | Vulval cancer | 78 | 9.783 | 457.364 |
| Other and unspecified genital organs | Choriocarcinoma | 7 | 4.682 | 14.405 |
| Other and unspecified genital organs | Pelvic neoplasm | 30 | 6.02 | 100.381 |
| Haematologic | Haematological malignancy | 1686 | 104.639 | 38646.36 |
| Haematologic | Haematopoietic neoplasm | 75 | 30.101 | 1040.063 |
| Haematologic | Splenic neoplasm malignancy unspecified | 31 | 18.297 | 304.246 |
| Haematologic | Malignant splenic neoplasm | 25 | 23.516 | 289.662 |
| Haematologic | Systemic mastocytosis | 22 | 5.214 | 60.214 |
| Haematologic | Benign spleen tumour | 18 | 30.101 | 238.397 |
| Haematologic | Follicular dendritic cell sarcoma | 7 | 70.235 | 122.667 |
| Haematologic | Chronic lymphocytic leukaemia stage 1 | 6 | 30.101 | 70.038 |
| Haematologic | Lymphocytic leukaemia | 16 | 2.384 | 10.627 |
| Haematologic | Leukaemia | 944 | 5.916 | 3220.345 |
| Lymphomas | Follicular lymphoma | 24 | 10.175 | 141.397 |
| Lymphomas | Gastrointestinal lymphoma | 15 | 5.131 | 39.053 |
| Lymphomas | Lymphoma | 1049 | 3.951 | 2042.713 |
| Lymphomas | Metastatic lymphoma | 47 | 8.523 | 237.198 |
| Nervous system | Brain neoplasm malignant | 1964 | 52.178 | 36057.78 |
| Nervous system | Brain cancer metastatic | 176 | 23.134 | 2094.668 |
| Nervous system | Brain stem glioma | 28 | 13.594 | 216.18 |
| Nervous system | Spinal cord neoplasm | 50 | 3.735 | 86.575 |
| Nervous system | Malignant neoplasm of spinal cord | 17 | 6.823 | 64.053 |
| Nervous system | Neuroblastoma | 20 | 3.562 | 30.635 |
| Nervous system | Aesthesioneuroblastoma | 3 | 8.209 | 9.645 |
| Head and neck | Malignant neoplasm of eye | 23 | 5.722 | 71.261 |
| Head and neck | Ear neoplasm malignant | 15 | 5.19 | 39.663 |
| Head and neck | Nasal cavity cancer | 414 | 75.526 | 8654.605 |
| Head and neck | Nasal sinus cancer | 36 | 14.258 | 291.946 |
| Head and neck | Nasal neoplasm | 35 | 9.755 | 200.927 |
| Head and neck | Sinus cancer metastatic | 3 | 5.017 | 5.095 |
| Head and neck | Head and neck cancer | 76 | 6.518 | 287.155 |
| Head and neck | Squamous cell carcinoma of head and neck | 11 | 3.989 | 19.115 |
| Skin | Skin cancer | 4356 | 17.176 | 42284.82 |
| Skin | Malignant sweat gland neoplasm | 4 | 5.734 | 9.344 |
| Bone | Bone cancer | 1167 | 21.161 | 13153.839 |
| Bone | Bone cancer metastatic | 66 | 10.401 | 409.629 |
| Bone | Ewing's sarcoma | 11 | 2.057 | 4.614 |
| Bone | Chondrosarcoma | 10 | 3.541 | 14.052 |
| Soft tissue | Leiomyosarcoma | 39 | 6.747 | 151.14 |
| Soft tissue | Liposarcoma | 17 | 3.12 | 20.251 |
| Soft tissue | Chest wall tumour | 6 | 4.013 | 9.389 |
| Soft tissue | Connective tissue neoplasm | 3 | 5.017 | 5.095 |
| Soft tissue | Sarcoma | 60 | 3.015 | 71.573 |
| Soft tissue | Soft tissue sarcoma | 15 | 2.415 | 10.194 |
| Soft tissue | Sarcoma metastatic | 6 | 2.617 | 4.087 |
| Soft tissue | Synovial sarcoma | 6 | 3.408 | 7.07 |
| Soft tissue | Mesothelioma | 26 | 2.756 | 25.009 |
| Mediastinal | Thymoma | 22 | 4.979 | 56.554 |
| Mediastinal | Neoplasm of thymus | 7 | 10.535 | 37.749 |
| Mediastinal | Thymoma malignant | 6 | 5.017 | 13.174 |
| Mediastinal | Malignant neoplasm of thorax | 5 | 5.574 | 12.099 |
| Mediastinal | Malignant neoplasm of thymus | 5 | 7.525 | 17.56 |
| Mediastinal | Thymic cancer metastatic | 4 | 6.689 | 11.391 |
| Cardiovascular | Cardiac neoplasm malignant | 15 | 15.569 | 125.049 |
| Cardiovascular | Cardiac neoplasm unspecified | 12 | 3.376 | 15.902 |
| Site unspecified | Neoplasm malignant | 10373 | 9.337 | 59158.39 |
| Site unspecified | Adenocarcinoma | 92 | 2.369 | 66.171 |
| Site unspecified | Adenoid cystic carcinoma | 25 | 11.577 | 166.68 |
| Site unspecified | Small cell carcinoma | 25 | 5.085 | 66.615 |
| Site unspecified | Malignant neoplasm of unknown primary site | 13 | 2.525 | 9.637 |
| Site unspecified | Malignant polyp | 8 | 3.884 | 12.649 |
| Site unspecified | Basosquamous carcinoma | 7 | 3.398 | 8.537 |
| Site unspecified | Mucoepidermoid carcinoma | 7 | 3.01 | 6.758 |
| Site unspecified | Mixed adenoneuroendocrine carcinoma | 4 | 40.135 | 49.235 |
| Site unspecified | Adenocarcinoma metastatic | 3 | 4.753 | 4.694 |

AEs, adverse events; PTs, Preferred Terms; PRR, proportional reporting ratio; χ^2^, chi-square.
