# Supplementary material for: Cancer related adverse events associated with use of proton pump inhibitors and histamine-2 receptor antagonists: A real-world analysis using the FDA adverse event reporting system
Source: PLoS One. 2025 Aug 12;20(8):e0329385. doi: 10.1371/journal.pone.0329385 (PMC12342331; doi:10.1371/journal.pone.0329385)
Supplement: S9 Table — (DOCX) [file pone.0329385.s009.docx]

**Supplementary Table 9.** Cancer related AEs with positive signals for H2RAs as a class (except ranitidine).

| **Cancer site** | **PTs** | **N** | **PRR** | **χ^2^** |
| --- | --- | --- | --- | --- |
| Gastric | Adenocarcinoma gastric | 11 | 11.063 | 87.746 |
| Gastric | Metastatic gastric cancer | 3 | 5.184 | 6.265 |
| Intestinal | Rectal cancer | 17 | 3.244 | 23.944 |
| Pancreatic | Pancreatic carcinoma metastatic | 10 | 2.486 | 7.399 |
| Lip and oral cavity | Malignant palate neoplasm | 3 | 27.599 | 48.276 |
| Lip and oral cavity | Tongue neoplasm | 4 | 9.066 | 20.588 |
| Lung | Lung adenocarcinoma | 16 | 3.186 | 21.632 |
| Lung | Lung neoplasm | 28 | 2.522 | 24.022 |
| Breast | Breast cancer male | 4 | 4.566 | 7.738 |
| Prostatic | Neoplasm prostate | 3 | 4.965 | 5.845 |
| Uterine and cervix | Cervix carcinoma | 18 | 2.122 | 9.523 |
| Haematologic | Marrow hyperplasia | 12 | 10.514 | 90.93 |
| Haematologic | Splenic neoplasm malignancy unspecified | 3 | 11.878 | 19.239 |
| Haematologic | Plasmacytoma | 11 | 3.773 | 19.486 |
| Haematologic | Transformation to acute myeloid leukaemia | 4 | 4.452 | 7.419 |
| Lymphomas | Lymphatic system neoplasm | 4 | 18.674 | 47.517 |
| Lymphomas | Extranodal marginal zone B-cell lymphoma (MALT type) | 5 | 5.213 | 12.847 |
| Lymphomas | Metastatic lymphoma | 7 | 10.629 | 50.081 |
| Nervous system | Glioblastoma multiforme | 10 | 6.503 | 40.372 |
| Nervous system | Malignant cranial nerve neoplasm | 7 | 437.899 | 1095.315 |
| Head and neck | Retro-orbital neoplasm | 7 | 91.229 | 416.085 |
| Skin | Malignant melanoma stage II | 3 | 6.516 | 8.838 |
| Soft tissue | Inflammatory myofibroblastic tumour | 3 | 13.799 | 22.921 |
| Mediastinal | Thymoma | 3 | 6.173 | 8.173 |

AEs, adverse events; H2RAs, histamine-2 receptor antagonists; PTs, Preferred Terms; PRR, proportional reporting ratio; χ^2^, chi-square.
