# Supplementary material for: Cancer related adverse events associated with use of proton pump inhibitors and histamine-2 receptor antagonists: A real-world analysis using the FDA adverse event reporting system
Source: PLoS One. 2025 Aug 12;20(8):e0329385. doi: 10.1371/journal.pone.0329385 (PMC12342331; doi:10.1371/journal.pone.0329385)
Supplement: S10 Table — (DOCX) [file pone.0329385.s010.docx]

**Supplementary Table 10.** Cancer related AEs with positive signals for Cimetidine.

| **Cancer site** | **PTs** | **N** | **PRR** | **χ^2^** |
| --- | --- | --- | --- | --- |
| Gastric | Adenocarcinoma gastric | 3 | 25.058 | 46.876 |
| Intestinal | Rectal cancer | 4 | 6.452 | 13.346 |
| Lip and oral cavity | Malignant palate neoplasm | 3 | 235.101 | 445.431 |
| Lip and oral cavity | Tongue neoplasm | 3 | 57.507 | 112.415 |
| Lung | Non-small cell lung cancer | 4 | 6.204 | 12.614 |
| Lung | Lung cancer metastatic | 6 | 9.596 | 37.871 |
| Thyroid | Parathyroid tumour | 5 | 6.928 | 19.73 |
| Breast | Breast neoplasm | 3 | 12.648 | 21.482 |
| Lymphomas | Lymphatic system neoplasm | 4 | 159.073 | 453.087 |
| Site unspecified | Adenocarcinoma | 4 | 8.479 | 19.376 |

AEs, adverse events; PTs, Preferred Terms; PRR, proportional reporting ratio; χ^2^, chi-square.
