# Supplementary material for: Cancer related adverse events associated with use of proton pump inhibitors and histamine-2 receptor antagonists: A real-world analysis using the FDA adverse event reporting system
Source: PLoS One. 2025 Aug 12;20(8):e0329385. doi: 10.1371/journal.pone.0329385 (PMC12342331; doi:10.1371/journal.pone.0329385)
Supplement: S11 Table — (DOCX) [file pone.0329385.s011.docx]

**Supplementary Table 11.** Cancer related AEs with positive signals for famotidine.

| **Cancer site** | **PTs** | **N** | **PRR** | **χ^2^** |
| --- | --- | --- | --- | --- |
| Gastric | Adenocarcinoma gastric | 10 | 11.528 | 83.216 |
| Gastric | Metastatic gastric cancer | 3 | 5.961 | 7.783 |
| Intestinal | Rectal cancer | 13 | 2.846 | 13.662 |
| Pancreatic | Pancreatic carcinoma metastatic | 10 | 2.859 | 10.216 |
| Lung | Lung adenocarcinoma | 16 | 3.663 | 28.083 |
| Lung | Lung neoplasm | 24 | 2.483 | 19.661 |
| Lung | Non-small cell lung cancer metastatic | 4 | 4.611 | 7.88 |
| Adrenal | Adrenal neoplasm | 3 | 4.199 | 4.403 |
| Breast | Breast cancer male | 4 | 5.251 | 9.691 |
| Prostatic | Neoplasm prostate | 3 | 5.709 | 7.293 |
| Uterine and cervix | Cervix carcinoma | 18 | 2.44 | 13.801 |
| Haematologic | Marrow hyperplasia | 10 | 10.019 | 70.435 |
| Haematologic | Splenic neoplasm malignancy unspecified | 3 | 13.659 | 22.781 |
| Haematologic | Transformation to acute myeloid leukaemia | 4 | 5.12 | 9.319 |
| Lymphomas | Metastatic lymphoma | 7 | 12.222 | 59.299 |
| Lymphomas | Diffuse large B-cell lymphoma stage III | 3 | 28.396 | 50.234 |
| Lymphomas | Extranodal marginal zone B-cell lymphoma (MALT type) | 5 | 5.995 | 15.837 |
| Lymphomas | Plasmacytoma | 11 | 4.338 | 24.712 |
| Nervous system | Glioblastoma multiforme | 7 | 5.202 | 19.452 |
| Nervous system | Malignant cranial nerve neoplasm | 7 | 503.547 | 1260.412 |
| Head and neck | Retro-orbital neoplasm | 7 | 104.906 | 479.986 |
| Skin | Malignant melanoma stage II | 3 | 7.493 | 10.772 |
| Soft tissue | Inflammatory myofibroblastic tumour | 3 | 15.868 | 27.022 |
| Mediastinal | Thymoma | 3 | 7.099 | 10.001 |

AEs, adverse events; PTs, Preferred Terms; PRR, proportional reporting ratio; χ^2^, chi-square.
