# Supplementary material for: Cancer related adverse events associated with use of proton pump inhibitors and histamine-2 receptor antagonists: A real-world analysis using the FDA adverse event reporting system
Source: PLoS One. 2025 Aug 12;20(8):e0329385. doi: 10.1371/journal.pone.0329385 (PMC12342331; doi:10.1371/journal.pone.0329385)
Supplement: S14 Table — (DOCX) [file pone.0329385.s014.docx]

**Supplementary Table 14.** Number of cancer related PTs with positive signals in each cancer site for H2RAs.

| **Cancer site** | **Ranitidine** | **H2RAs as a class (except ranitidine)** | **Cimetidine** | **Famotidine** | **Nizatidine** |
| --- | --- | --- | --- | --- | --- |
| Gastric | 9 | 2 | 1 | 2 | 0 |
| Intestinal | 13 | 1 | 1 | 1 | 0 |
| Pancreatic | 1 | 1 | 0 | 1 | 0 |
| Oesophageal | 3 | 0 | 0 | 0 | 0 |
| Hepatobiliary | 7 | 0 | 0 | 0 | 0 |
| abdominal wall and peritoneal | 2 | 0 | 0 | 0 | 0 |
| Lip and oral cavity | 7 | 2 | 2 | 0 | 0 |
| Anal canal | 2 | 0 | 0 | 0 | 0 |
| Upper respiratory tract | 10 | 0 | 0 | 0 | 0 |
| Lung | 2 | 2 | 2 | 3 | 0 |
| Thyroid | 2 | 0 | 1 | 0 | 0 |
| Adrenal | 2 | 0 | 0 | 1 | 0 |
| Other and unspecified endocrine glands | 4 | 0 | 0 | 0 | 0 |
| Renal | 5 | 0 | 0 | 0 | 0 |
| Ureteric | 2 | 0 | 0 | 0 | 0 |
| Bladder | 2 | 0 | 0 | 0 | 0 |
| Urinary tract | 3 | 0 | 0 | 0 | 0 |
| Breast | 6 | 1 | 1 | 1 | 0 |
| Prostatic | 3 | 1 | 0 | 1 | 0 |
| Testicular | 3 | 0 | 0 | 0 | 0 |
| Penile | 1 | 0 | 0 | 0 | 0 |
| Ovarian and fallopian tube | 3 | 0 | 0 | 0 | 0 |
| Uterine and cervix | 4 | 1 | 0 | 1 | 0 |
| Vulvovaginal | 2 | 0 | 0 | 0 | 0 |
| Other and unspecified genital organs | 2 | 0 | 0 | 0 | 0 |
| Haematologic | 10 | 4 | 0 | 3 | 0 |
| Lymphomas | 4 | 3 | 1 | 4 | 0 |
| Nervous system | 7 | 2 | 0 | 2 | 0 |
| Head and neck | 8 | 1 | 0 | 1 | 0 |
| Skin | 2 | 1 | 0 | 1 | 0 |
| Bone | 4 | 0 | 0 | 0 | 0 |
| Soft tissue | 9 | 1 | 0 | 1 | 0 |
| Mediastinal | 6 | 1 | 0 | 1 | 0 |
| Cardiovascular | 2 | 0 | 0 | 0 | 0 |
| Site unspecified | 10 | 0 | 1 | 0 | 1 |

PTs: Preferred Terms; H2RAs, histamine-2 receptor antagonists.
